# Age-Associated Changes in Adverse Events Arising From Anti-PD-(L)1 Therapy

**DOI:** 10.3389/fonc.2021.619385

**Published:** 2021-05-13

**Authors:** Xinyi Huang, Tiantian Tian, Yan Zhang, Shengjian Zhou, Pingping Hu, Jiandong Zhang

**Affiliations:** ^1^ Department of Oncology, Shandong Provincial Qianfoshan Hospital, Cheeloo College of Medicine, Shandong University, Jinan, China; ^2^ Department of Oncology, The First Affiliated Hospital of Shandong First Medical University & Shandong Provincial Qianfoshan Hospital, Jinan, China; ^3^ Shandong Lung Cancer Institute, Jinan, China; ^4^ Department of Oncology, Liangshan County People’s Hospital, Jining, China

**Keywords:** aging, immune checkpoint inhibitors, immune-related adverse events, immune cell infiltration, lung cancer

## Abstract

**Background:**

Immune-related adverse events (irAEs) may complicate the immune checkpoint inhibition (ICI) therapy. The effect of age on these irAEs is not elucidated. The aim of the study was to compare the occurrence of irAEs in different age groups.

**Methods:**

Patients with lung cancer receiving anti-programmed death- (ligand)1 (PD-(L)1) were selected from the US Food and Drug Administration Adverse Event Reporting System (FAERS) database. Immune cell infiltration data set was obtained from TIMER 2.0 web server. The patients were stratified for age as follows: <65 year-old (young patients, YP), 65 to 75 year-old (middle aged patients, MP), ≥75 year-old (old patients, OP). The severity of irAEs was compared using logistic binary regression model. The distribution differences of immune cell infiltration were estimated using non-parametric tests.

**Results:**

Of all the 17,006 patients treated by anti-PD-(L)1, 7,355 were <65 (YP), 6,706 were 65–75 (MP), and 2,945 were ≥75 (OP). In general, we analyzed a total of 16 irAEs in this article and found that pulmonary toxicity was more frequent in OP (OP *vs.* YP: OR = 1.45, 95% CI: 1.28–1.64) and MP (MP *vs.* YP: OR = 1.38, 95% CI: 1.24–1.52), but hepatitis was less frequent in OP (OP *vs.* YP: OR = 0.56, 95% CI: 0.32–0.97) and MP (MP *vs.* YP: OR = 0.57, 95%CI: 0.38–0.85). Further analysis demonstrated that older patients showed less B cell, CD8^+^ T cell and myeloid dendritic cell infiltration than younger patients.

**Conclusions:**

Elderly patients exhibited higher incidences of pulmonary toxicity, while hepatitis was found at low incidence. Therefore, clinicians should carefully monitor comorbidities in elderly patients.

## Introduction

Programmed cell death protein-1 (PD-1) and programmed cell death protein- ligand 1 (PD-L1) are the two most intensively studied immune regulatory checkpoint pathways in cancer (1), which relies on the presence of ongoing antitumor immune response after blocking this pathway (2). Monoclonal antibody therapies at various clinical levels have now been developed to against these immune checkpoint proteins (3, 4). Immune checkpoint inhibitors (ICIs) against PD-(L)1 have changed the treatment landscape of many different cancers so far. Responses occur in a large proportion of patients and are often long-lasting and even curative (5, 6). PD-(L)1 inhibitors can reactivate previously activated T cells that have lost their effector and proliferative functions during the process of immune response. Potential host anti-tumor immune response is the basis for the clinical benefit of PD-(L)1 agents (7).

Although ICIs such as anti-PD-1 or anti-PD-L1 have been shown to be effective against many cancers, patients who receive ICIs may experience immune-related adverse events (irAEs). IrAEs are common side effects of checkpoint inhibition (CPI) therapy for PD-(L)1. It has been found that the toxic effects associated with ICIs may occur at any part of the body and result from the activation of autoreactive T cells, thereby destroying host tissues (8). The most representative irAEs are usually colitis, hepatitis, pneumonia, hypophysitis, thyroid toxicity, and skin toxicity, and adverse events involving the heart, nervous system, and other organs, though rare, can also occur. These rare, violent, and deadly toxic effects may complicate the transformative treatment of PD-(L)1. These toxic effects are a major clinical challenge and an obstacle to the development of more effective combinations (9).

ICIs has now showed noteworthy therapeutic advantages compared with traditional therapies. However, there is still relatively limited information on the use of ICIs and the irAEs generated by ICIs in older patients. Previous studies found that the body’s immune system function declines with age, manifested by a higher tendency to respond to autoantigens, a decrease in the ability of host defenses against microbes and cancer, and disorders between different immune system components. These signs of a weakened immune system may be associated with “immunosenescence,” which may reduce the efficacy and safety of immune-based therapies and may contribute to the increased incidence of irAEs and development of cancer (10, 11). As only a small part of the participants are 75 years or older, the representativeness of the elderly population in clinical trials is generally low. Research on the irAEs of elderly is sparse. In this study, we use two large real-world data sets to explore the differences of irAEs and determine the distribution differences of tumor-infiltrating immune cells among patients of different ages.

## Methods

### Data Selection and Preprocessing

The US Food and Drug Administration Adverse Event Reporting System (FAERS) is a database designed to support the Food and Drug Administration (FDA)’s post-marketing monitoring program for drugs and therapeutic biological products. The database includes all adverse event information and medication error information. In this study, we extracted 17006 eligible lung cancer (LC) patients receiving PD-(L)1 inhibitor treatment registered as of December 31, 2019 from FAERS. The study was exempt from ethical review under the EKOS (Ethikkommission Ostschweiz, Switzerland) ethics committee policy because all of the analyzed data sets were identified and publicly available. Five reported PD-(L)1 monoclonal antibodies were searched from FAKERS public dashboard. Search terms included “nivolumab,” “pembrolizumab,” “atezolizumab,” “avelumab,” and “durvalumab.” We also selected sixteen common irAEs, including pulmonary toxicity, radiation pneumonitis, myasthenia gravis, adrenal insufficiency, colitis, myocarditis, hepatitis, myositis, hypophysitis, encephalitis, skin reaction, diabetes, thyroid toxicity, hematologic toxicity, neurologic toxicity, and gastrointestinal reaction (9). Clinicopathological characteristics enrolled in the model were sex, serious, pathological type, and country. Serious means that one or more of the following outcomes were documented in the report: death, hospitalization, life-threatening, disability, congenital anomaly, and/or other serious outcome. Subsequently, the cohort was trichotomized into three (younger patients (YP) with age <65, middle aged patients (MP) with 65≤age<75, and elder patients with age≥75) subgroups using cut-off age of 65 and 75 years.

### Tumor-Infiltrating Immune Cell Analysis

The data set of tumor-infiltrating immune cells was downloaded from TIMER 2.0 (http://timer.cistrome.org). TIMER 2.0 consists of three major components, including immune, exploration, and estimation. The estimation component was used to infer immune cell infiltration levels. The TIMER algorithm was chosen for our study. Then, the TIMER data set was matched with the lung cancer data set obtained from the Cancer Genome Atlas (TCGA) database (https://portal.gdc.cancer.gov) according to the TCGA ID number. Similarly, the immune infiltration data was stratified into three subgroups by age (<65 years, 65–75 years, and ≥75 years). A violin plot was constructed using Origin ver. 2019 to show the distribution of tumor-infiltrating immune cells.

### Statistical Analysis

All data manipulation and statistical analyses were performed using IBM SPSS version 22 (IBM Corporation, Armonk, NY, USA) and Microsoft Excel (2016, Microsoft). The differences in irAEs of each age group were calculated using a crosstab. Univariate logistic binary regression analysis was applied to calculate the odds ratio (ORs) and 95% confidence intervals (CIs). Subsequently, multivariate logistic regression was used to estimate the odds ratios (ORs) and 95% CI for the association between age and different irAEs, while controlling for potential confounders, including sex, treatment modality, comorbidity (pre-existing autoimmune condition). A forest plot was generated using Stata ver.12.0 to summarize data for each group with ORs and 95% CIs to provide a visual analysis of studies evaluating fatal toxicity events. The distribution of immune cells among different age groups was analyzed using TIMER 2.0 web server. Statistically significant difference was defined as a P-value <0.05.

### Ethical Statement

The authors are accountable for all aspects of the work in ensuring that questions related to the accuracy or integrity of any part of the work are appropriately investigated and resolved. Institutional review board approval was not required because FAERS is an unlinkable anonymized database open to the public. Informed consents from patients were waived due to the anonymity of individual patient data. The study was conducted in accordance with the Declaration of Helsinki (as revised in 2013).

## Results

### Characteristics of the Study Population

In our study, we identified 17006 LC cases from FDA database. Among them, 7355 (43.2%) patients were in the YP (age <65) group, 6706 (39.4%) were in the MP (65≤age<75) group and 2945 (17.4%) were in the OP (age≥75) group. The median (range) age of YP, MP, and OP subgroups was respectively 58 (0–64), 69 (65–74), and 78 (75–101) years old. In total, 11335 (66.7%) of the patients were male, 10584 (62.2%) were non-small cell lung cancer, and 16351 (96.1%) had serious outcomes. The main countries were Japan (5826[34.3%]), United States (3506[20.6%]), France (1881[11.1%]), and others (1562[9.2%]). The baseline characteristics in each subgroup are presented in **Table 1**.

**Table 1:** Characteristics of 17006 Patients.

Characteristics	All n=17006	age＜65 n=7355	65≤age<75 n=6706	age≥75 n=2945	P-value
**Sex, No. (%)**					<0.001
Female	5414 (31.8)	2660 (36.2)	1895 (28.3)	859 (29.2)	
Male	11335 (66.7)	4582 (62.3)	4723 (70.4)	2030 (68.9)	
Not specified	257 (1.5)	113 (1.5)	88 (1.3)	56 (1.9)	
**Serious, No. (%)**					<0.001
Serious	16351 (96.1)	7074 (96.2)	6485 (96.7)	2792 (94.8)	
Non-serious	655 (3.9)	281 (3.8)	221 (3.3)	153 (5.2)	
**Pathological type, No. (%)**					<0.001
Non-small cell lung cancer	10584 (62.2)	4477 (60.9)	4151 (61.9)	1956 (66.4)	
Small cell lung cancer	631 (3.7)	316 (4.3)	219 (3.3)	96 (3.3)	
Not specified	5791 (34.1)	2562 (34.8)	2336 (34.8)	893 (30.3)	
**Country, No. (%)**					<0.001
Japan	5826 (34.3)	1906 (25.9)	2663 (39.7)	1257 (42.7)	
United States	3506 (20.6)	1598 (21.7)	1240 (18.5)	668 (22.7)	
France	1881 (11.1)	972 (13.2)	660 (9.8)	249 (8.5)	
Germany	963 (5.7)	504 (6.9)	340 (5.1)	119 (4.0)	
Italy	632 (3.7)	227 (3.1)	279 (4.2)	126 (4.3)	
Spain	414 (2.4)	220 (3.0)	144 (2.1)	50 (1.7)	
China	366 (2.2)	235 (3.2)	108 (1.6)	23 (0.8)	
United Kingdom	312 (1.8)	125 (1.7)	132 (2.0)	55 (1.9)	
Australia	300 (1.8)	120 (1.6)	121 (1.8)	59 (2.0)	
Canada	296 (1.7)	135 (1.8)	114 (1.7)	47 (1.6)	
Belgium	229 (1.3)	114 (1.5)	90 (1.3)	25 (0.8)	
Republic of Korea	142 (0.8)	66 (0.9)	62 (0.9)	14 (0.5)	
Netherlands	137 (0.8)	70 (1.0)	55 (0.8)	12 (0.4)	
Switzerland	117 (0.7)	55 (0.7)	42 (0.6)	20 (0.7)	
India	115 (0.7)	80 (1.1)	27 (0.4)	8 (0.3)	
Israel	108 (0.6)	46 (0.6)	37 (0.6)	25 (0.8)	
Brazil	100 (0.6)	49 (0.7)	41 (0.6)	10 (0.3)	
Others	1562 (9.2)	833 (11.3)	551 (8.2)	178 (6.0)	

Serious means that one or more of the following outcomes were documented in the report: death, hospitalization, life-threatening, disability, congenital anomaly, and/or other serious outcomes. Documenting one or more of these outcomes in a report does not necessarily mean that the suspect product(s) named in the report was the cause of the outcomes.

We run an univariate and multivariate logistic regression analysis of the odds ratio for different irAEs (**Supplementary Table 1**). In the univariate analysis, the incidence of irAEs including pulmonary toxicity, radiation pneumonitis, hepatitis, hypophysitis, hematologic toxicity, and gastrointestinal reaction was significantly higher for OP than MP and YP, and subjects with pulmonary toxicity, radiation pneumonitis, adrenal insufficiency, encephalitis, skin reaction, hematologic toxicity were more likely to be males. Multivariate analysis, after controlling for the confounders, demonstrated an independent and significant association between demographic and clinical characteristics and the increased likelihood of irAEs (**Supplementary Table 1**). The risk of pulmonary toxicity was independently positively associated with older subjects [adjusted OR of 1.381 (95% CI 1.243–1.534, p<0.001) and being male [adjusted OR of 1.537 (95% CI 1.407–1.680, p<0.001); adjusted OR of 1.418 (95% CI 1.115–1.804, p=0.004)]. In addition, patients receiving combinational agent treatment [adjusted OR of 1.334 (95% CI 1.236–1.441, p<0.001)] were also observed with increased pulmonary toxicity. Independent negative associations were observed among the risk of combinational agent treatment [adjusted OR of 0.652 (95% CI 0.526–0.808, p<0.001] (**Supplementary Table 1**). However, subjects who have hepatitis were younger [adjusted OR of 0.622 (95% CI 0.420–0.922, p=0.018)] and received combinational agents [adjusted OR of 2.924 (95% CI 1.966–4.349, p<0.001)].

### Impact of Aging on Immune-Related Adverse Events

To confirm whether aging increases the risk of irAEs, we performed analyses of the association between age and irAEs using a crosstab. A total of 16 irAEs were included in our analysis (**Table 2**, **Supplementary Table 1**, P<0.05). Among 2137 (12.6%) patients with pulmonary toxicity, 772 (10.5%) were in the YP group, 939 (14%) were in the MP group, and 426 (14.5%) were in the OP group. Compared with YP, OP (OP vs. YP: adjusted OR = 1.381, 95% CI: 1.243–1.534; P<0.001) and MP (MP vs. YP: adjusted OR = 1.270, 95% CI: 1.163–1.388; P<0.001) had increased risks of developing pulmonary toxicity. We also found that OP group had a higher risk of developing pulmonary toxicity than MP group. Within 207 (1.2%) patients with adrenal insufficiency and 177 (1.0%) with hematologic toxicity, the risk of developing adrenal insufficiency (MP *vs.* YP: adjusted OR = 1.505, 95% CI: 1.092–2.074) in the MP group and hematologic toxicity (OP *vs.* YP: adjusted OR = 1.513, 95% CI: 1.024–2.236) in the OP group were higher than that in the YP group, while the risk of developing gastrointestinal reaction (OP *vs.* YP: adjusted OR=1.537, 95% CI: 1.041–2.268) in the OP group was higher than that in the YP group. However, in the 124 (0.7%) patients with hepatitis, both the OP (OP vs. YP: adjusted OR=0.504, 95% CI: 0.295–0.861) group and the MP (MP *vs.* YP: adjusted OR=0.614, 95%CI: 0.416–0.907) group reduced the risk of irAEs. Besides, the OP group had a lower risk of developing hepatitis than MP group. The risk of other irAEs did not differ among the YP, MP, and OP group (**Table 2**, P > 0.05).

**Table 2:** Univariate and multivariate logistic regression analysis of the odds ratio for different irAEs, controlling for multiple conditions.

Variable	Category	Univariate			Multivariate			Univariate			Multivariate		
		Crude OR	95%CI	*p* value	Adjusted OR	95%CI	*p* value	Crude OR	95%CI	*p* value	Adjusted OR	95%CI	*p* value
		Pulmonary toxicity						Gastrointestinal reaction					
Age	65≤age<75	1.329	1.217–1.451	**<0.001**	1.270	1.163–1.388	**<0.001**	1.157	0.815–1.644	0.414	1.151	0.811–1.635	0.431
	≥75	1.414	1.274–1.570	**<0.001**	1.381	1.243–1.534	**<0.001**	1.527	1.035–2.253	**0.033**	1.537	1.041–2.268	**0.031**
Sex	Male	1.609	1.473–1.756	**<0.001**	1.537	1.407–1.680	**<0.001**	1.247	0.892–1.742	0.196			
Treatment modality	Combinational	1.334	1.236–1.441	**<0.001**	1.300	1.203–1.405	**<0.001**	1.503	1.110–2.035	**0.008**	1.509	1.114–2.044	**0.008**
Comorbidity	Yes	1.236	0.694–2.201	0.472				0.000	0.000	0.997			
		Myasthenia gravis						Adrenal insufficiency					
Age	65≤age<75	1.576	0.914–2.718	0.102				1.559	1.133–2.146	**0.006**	1.505	1.092–2.074	**0.012**
	≥75	3.031	1.751–5.246	**<0.001**				1.365	0.925–2.013	0.117	1.354	0.917–1.999	0.127
Sex	Male	0.795	0.516–1.226	0.299				1.411	1.028–1.936	**0.033**	1.341	0.976–1.843	0.070
Treatment modality	Combinational	0.768	0.502–1.177	0.226				1.481	1.123–1.953	**0.005**	1.432	1.084–1.892	**0.011**
Comorbidity	Yes	2.935	0.403–21.388	0.288				0.000	0.000	0.997			
		Colitis						Myocarditis					
Age	65≤age<75	1.103	0.920–1.323	0.289				1.248	0.898–1.733	0.187			
	≥75	1.079	0.865–1.345	0.501				1.018	0.670–1.547	0.933			
Sex	Male	1.011	0.851–1.202	0.897				0.886	0.651–1.205	0.441			
Treatment modality	Combinational	1.451	1.234–1.705	**<0.001**	1.433	1.219–1.684	**<0.001**	1.216	0.908–1.629	0.189			
Comorbidity	Yes	0.403	0.056–2.911	0.368				1.418	0.196–10.278	0.729			
		Hepatitis						Myositis					
Age	65≤age<75	0.610	0.412–0.904	**0.014**	0.614	0.416–0.907	**0.014**	1.230	0.857–1.767	0.262			
	≥75	0.499	0.292–0.852	**0.011**	0.504	0.295–0.861	**0.012**	1.488	0.988–2.242	0.057			
Sex	Male	0.921	0.633–1.338	0.665				1.450	1.012–2.076	0.043			
Treatment modality	Combinational	2.964	1.995–4.403	**<0.001**	2.924	1.966–4.349	**<0.001**	1.072	0.786–1.462	0.663			
Comorbidity	Yes	4.277	1.035–17.673	**0.045**	3.063	0.736–12.740	0.124	3.248	0.788–13.383	0.103			
		Hypophysitis						Encephalitis					
Age	65≤age<75	0.921	0.587–1.446	0.721				0.857	0.591–1.242	0.415			
	≥75	0.392	0.183–0.839	**0.016**				1.281	0.857–1.915	0.228			
Sex	Male	1.128	0.706–1.803	0.615				0.644	0.467–0.889	**0.007**	0.646	0.468–0.892	**0.008**
Treatment modality	Combinational	1.516	0.985–2.334	0.059				1.394	1.012–1.920	**0.042**	1.363	0.989–1.878	0.058
Comorbidity	Yes	0.000	0.000	0.997				0.000	0.000	0.997			
		Skin reaction						Diabetes					
Age	65≤age<75	1.092	0.847–1.409	0.496				1.226	0.942–1.595	0.130			
	≥75	0.957	0.695–1.319	0.790				1.121	0.811–1.550	0.488			
Sex	Male	0.708	0.560–0.894	**0.004**	0.721	0.571–0.911	**0.006**	1.219	0.941–1.579	0.135			
Treatment modality	Combinational	1.245	0.990–1.566	0.061				1.041	0.824–1.315	0.736			
Comorbidity	Yes	0.000	0.000	0.997				1.798	0.438–7.381	0.416			
		Thyroid toxicity						Hematologic toxicity					
Age	65≤age<75	0.848	0.723–0.994	**0.042**				0.802	0.650–0.991	**0.041**	1.130	0.795–1.607	0.496
	≥75	0.920	0.760–1.114	0.395				0.711	0.542–0.934	**0.014**	1.513	1.024–2.236	**0.037**
Sex	Male	0.877	0.756–1.017	0.083				0.818	0.670–0.998	**0.047**	1.228	0.878–1.719	0.231
Treatment modality	Combinational	0.996	0.864–1.148	0.955				1.503	1.110–2.035	**0.008**	1.511	1.115–2.047	**0.008**
Comorbidity	Yes	1.632	0.654–4.072	0.294				1.187	0.290–4.864	0.812			
		Neurologic toxicity											
Age	65≤age<75	0.795	0.329–1.919	0.609									
	≥75	0.174	0.022–1.348	0.094									
Sex	Male	1.194	0.463–3.080	0.713									
Treatment modality	Combinational	1.202	0.510–2.831	0.674									
Comorbidity	Yes	0.000	0.000	0.998									

irAEs, immune-related adverse events; Yes, with irAEs; No, without irAEs. Statistically significant values are in bold (p < 0.05).

In order to further explore the effect of irAEs on patients receiving anti-PD-(L)1 treatment in combination with anti-cytotoxic T lymphocyte-associated antigen-4 (CTLA-4) agents, we analyzed separately in OP, MP and YP subgroups. In YP subject treated with both anti-PD-(L)1 and anti-CTLA-4, 125 (4.0%) patients developed colitis, 49 (1.6%) developed hepatitis, 23 (0.7%) developed hypophysitis, 45 (1.4%) developed diabetes, implying an increased risk of irAEs with the combination treatment (**Table 3**, p<0.05). Among OP subjects, 29 (0.9%), 24 (0.7%), and 28 (0.8%) cases had an increased risk of developing hepatitis, hypophysitis, and encephalitis (**Table 3**, p<0.05). However, the risk of irAEs on patients aged 75 and older appeared to have no differences in treatment type.

**Table 3:** The severity of irAEs in patients with/without anti-CTLA4 agents.

irAEs	Age<65		65≤age<75		Age≥75	
	Combinational agents		Combinational agents		Combinational agents	
	OR (95%CI)	P-value	OR (95%CI)	P-value	OR (95%CI)	P-value
Pulmonary toxicity	0.900 (0.701–1.155)	0.407	0.776 (0.596–1.011)	0.060	0.898 (0.597–1.351)	0.605
Myasthenia gravis	0.590 (0.079–4.407)	0.607	1.512 (0.460–4.967)	0.495	0.693 (0.094–5.109)	0.719
Adrenal insufficiency	1.731 (0.820–3.653)	0.150	1.176 (0.542–2.553)	0.681	1.060 (0.254–4.423)	0.936
Colitis	1.981 (1.348–2.910)	**<0.001**	1.530 (0.985–2.375)	0.058	1.712 (0.852–3.440)	0.131
Myocarditis	0.579 (0.181–1.851)	0.357	1.443 (0.661–3.150)	0.357	2.108 (0.638–6.965)	0.221
Hepatitis	3.307 (1.818–6.016)	**<0.001**	3.154 (1.392–7.145)	**0.006**	2.994 (0.678–13.211)	0.148
Myositis	0.226 (0.031–1.637)	0.141	0.477 (0.116–1.955)	0.304	1.143 (0.273–4.779)	0.855
Hypophysitis	5.354 (2.696–10.632)	**<0.001**	3.563 (1.560–8.142)	**0.003**	3.194 (0.390–26.125)	0.279
Encephalitis	1.831 (0.865–3.876)	0.114	2.396 (1.075–5.340)	**0.033**	1.821 (0.555–5.974)	0.323
Skin reaction	0.531 (0.216–1.307)	0.168	0.493 (0.181–1.342)	0.166	0.000 (0.000–0.000)	0.996
Diabetes	2.067 (1.186–3.604)	**0.010**	1.182 (0.596–2.345)	0.633	1.616 (0.578–4.520)	0.360
Thyroid toxicity	0.799 (0.514–1.243)	0.320	0.868 (0.519–1.451)	0.589	0.262 (0.064–1.065)	0.061
Hematologic toxicity	0.363 (0.160–0.823)	0.015	0.588 (0.259–1.337)	0.205	0.301 (0.042–2.179)	0.234
Neurologic toxicity	0.000 (0.000–0.000)	0.993	0.000 (0.000–0.000)	0.994	0.000 (0.000–0.000)	0.997
Gastrointestinal reaction	0.635 (0.198–2.036)	0.444	0.225 (0.031–1.626)	0.139	0.000 (0.000–0.000)	0.996

irAEs, Immune-related adverse events; CTLA-4, cytotoxic T lymphocyte-associated antigen-4. Statistically significant values are in bold (p<0.05).

### The Distribution of Tumor-Infiltrating Immune Cells

**Figure 1:**
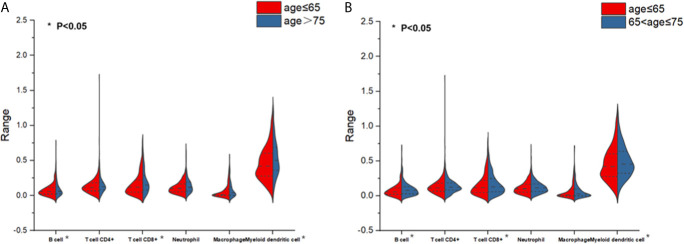
Split violin plots estimating the distributions and levels of immune cell infiltration. **(A)** Infiltration differences of 6 immune cells between patients aged ≥75 (old patients) versus patients aged <65 (young patients). Red indicates the young patient subgroup and blue indicates the old patient subgroup. **(B)** Infiltration differences of six immune cells between patients aged 65 to <75 (middle-aged patients) versus patients aged <65 (young patients). Red indicates the young patient subgroup and blue indicates the middle-aged patient subgroup.

To determine if aging affects tumor-associated immune cell infiltration as well as the number of immune cells primarily involved, split violin plots (**Figure 1**) were built, allowing a direct comparison between the two populations (OP and MP) and YP. As can be seen from **Figure 1A**, the immune cell infiltration level of B cell, CD8^+^ T cell, and myeloid dendritic cell in the OP group was significantly reduced compared with YP group. These immune cells infiltrated exhibited similar distribution patterns between groups of MP and YP (**Figure 1B**, P<0.05). CD8^+^ T cell, neutrophil, and macrophage infiltrated did not differ in the OP or MP group versus YP group. The statistical significance was lost in CD8^+^ T cell, neutrophil and macrophage. Therefore, the OP and MP groups may have unique biological features that are different from YP group.

## Discussion

ICI therapy is now increasingly used to treat a variety of solid tumors, including LC. However, the use of PD-(L)1 pathway inhibitors, such as monoclonal antibodies against PD-1 or PD-L1, will inevitably generate a variety of adverse events. ICIs have their own idiosyncratic adverse events, collectively defined as irAEs. Although ICI has a safe toxicity profile in cancer treatment, the toxicity of these molecules may be more challenging in elderly patients due to reduced functional reserve, age-associated comorbidities and polypharmacy.

Some clinical trials have found the relationship between age and toxicities. A previous clinical trial analyzing pooled data from a Nivolumab Phase III registry of different cancer types found that patients aged 70 years and older had higher skin toxicities than those under 65 years old (12). There was also an increase rate of grade III to V toxicities in patients aged 70 years or over than those under 70 years of age (13). In addition, a research team from Sloan Kettering Cancer Center presented at the American Society of Clinical Oncology (ASCO) meeting the benefits and toxicity of ICIs in patients over 80 years of age for melanoma (14). They reported that older patients had slightly higher rates of irAEs and early discontinuation of treatment than younger people. Our comprehensive study that included 17006 patients from FDA database investigated the occurrence of irAEs in elderly patients with lung cancer as compared with younger patients and middle-aged patients. In our results, our analysis showed an increased level of toxicities in older patients than in their younger counterparts when treated with anti-PD-(L)1 agents. Besides, the OP subgroup was having a higher risk of irAEs than MP and YP subgroup. Toxicities were more frequent on lung and endocrine in OP and MP compared with YP (**Table 2**). These results showed strong evidence of the increasing toxicities of anti-PD-(L)1 for older patients.

In recent years, more and more researchers have reached a consensus that immunosenescence has become a vital intersection of the increasing frequency and severity of cancer, aging, and immunity (15). Immunosenescence refers to a phenomenon of decreased immune function as a result of age-associated declines and impairments of immune function, affecting the process of producing specific responses to foreign antigens and autoantigens (16). One of the major theories to explain immunosenescence is autoimmunity (17). With advancing age, the immune system’s ability to distinguish between invaders and normal tissue diminishes and immune cells begin to attach normal body tissues. Similarly, irAEs are associated with infiltration of normal tissue by activated T cells responsible for autoimmunity. Autoimmune diseases caused by ICIs may be typical examples (18, 19).

T cells play an important role in anti-cancer immune defense mechanisms and they recognize tumor antigens, so they are activated and widely clear tumor cells. Studies have shown that diminished T-cell mediated immunity is the primary factor involved in the pathophysiology of immunosenescence (20). T cells undergo significant changes with aging: their absolute number, especially the naive CD8^+^T cells, declines with aging, partly due to thymic retreat and lymphoid stem cell contraction (21–23), and thus resulting in a decreased T cell diversity, decreased T cell proliferation and survival after T cell receptor stimulation, altered cytokines, and decreased cytotoxicity of CD8^+^T cells (24, 25). In this article, we explored the age-related immune cell alterations. Our results identified that older age is associated with less CD8^+^ T cell. Therefore, the decrease in the number and function of CD8^+^ T cells might lead to poor immunity in patients, which are more likely to have irAEs when using ICIs and thus have a direct impact on the efficacy and toxicity of ICIs in this population. Our current understanding of immunosenescence implicates changes in the adaptive immune system—particularly within T cell populations—as the primary determinants of declining immune function with age. On the other hand, with the increase of age, the infiltration of immune cells into normal tissues increases, which leads to immune hyperactivity and triggers autoimmunity, thereby potentially increasing the incidence of irAEs (17–19).

At the same time, irAEs may be more challenging in older patients due to reduced functional reserve and age-associated comorbidities. Therefore, early detection of irAEs should be strengthened for management of elderly patients, and the severity of irAEs should also be carefully monitored and evaluated as associated comorbidities may be more likely to be decompensated. Finally, it is well known that older patients have a higher prevalence of autoantibodies, and it is expected that ICIs may reveal subclinical autoimmune diseases. Therefore, it is important to investigate individual or familial autoimmune diseases or viral infections before ICI treatment to prevent irAEs.

This study has some limitations that warrant mention. First, the present study was a retrospective study. Second, adverse events reported in the FAERS database cannot be identified whether they were caused by the drug. When submitting the reports, FDA does not require proof of a causal relationship between an adverse event and a drug, and reports typically do not include detailed information that evaluates an adverse event. Third, the information stored in the FAERS database is basically based on spontaneous reporting. Whether an event can be reported is influenced by a variety of factors, such as the time the product is on the market and the level of public awareness of adverse events. FDA is unable to collect all serious adverse events from patients, which leads to reporting bias.

In conclusion, our study compared the risks of irAEs and the distribution differences of tumor-infiltrating immune cells among different age groups based on real-world data analyses. Our analysis showed increased pulmonary toxicity and decreased hepatitis toxicity in the older group than younger group. Less B cell, CD8^+^ T cell, and myeloid dendritic cell infiltration were observed in the patients aged ≥75 years. These trends often result in rapid clinical deterioration and poor outcomes. Therefore, clinicians should carefully assess and manage comorbidities in elderly patients, which is essential for better multidisciplinary cancer treatment.

## Data Availability Statement

Publicly available data sets were analyzed in this study. These data can be found here: The FAKERS data sets for this study can be found in the https://fis.fda.gov/sense/app/d10be6bb-494e-4cd2-82e4-0135608ddc13/sheet/6b5a135f-f451-45be-893d-20aaee34e28e/state/analysis. The TIMER 2.0 web server for this study can be found in http://timer.cistrome.org/.

## Ethics Statement

Institutional review board approval was not required because FAERS is an unlinkable anonymized database open to the public. Informed consents from patients were waived due to the anonymity of individual patient data. The study was conducted in accordance with the Declaration of Helsinki (as revised in 2013).

## Author Contributions

Conception and design: All authors. Administrative support: JZ. Provision of study materials or patients: All authors. Collection and assembly of data: PH, XH, TT, and YZ.Data analysis and interpretation: PH and XH. Manuscript writing: All authors. All authors contributed to the article and approved the submitted version.

## Funding

This study was funded by the National Natural Science Foundation of China (no. 81672974, 81803043, 81703033 and 81602719] and Shandong Natural Science Foundation [ZR2017BH042].

## Conflict of Interest

The authors declare that the research was conducted in the absence of any commercial or financial relationships that could be construed as a potential conflict of interest.

The reviewer XM declared a shared affiliation, with no collaboration, with one of the authors, XH, to the handling editor at the time of review.

## Supplementary Material

Supplementary Table 1 Association between patient characteristics and irAEs.
