# Supplementary material for: Age-Associated Changes in Adverse Events Arising From Anti-PD-(L)1 Therapy
Source: Front Oncol. 2021 May 13;11:619385. doi: 10.3389/fonc.2021.619385 (PMC8155669; doi:10.3389/fonc.2021.619385)
Supplement: Supplementary Table 1 — Association between patient characteristics and irAEs. [file Table_1.docx]

**Supplementary table 1 Association between patient characteristics and irAEs**.

| **IrAEs** **All**    **N=17006** | | | **Pulmonary toxicity** | |  | **Gastrointestinal reaction** | |  | **Myasthenia gravis** | |  | **Adrenal insufficiency** | |  | **Colitis** | |  | **Myocarditis** | |  | **Hepatitis** | |  | **Myositis** | |
| --- | --- | --- | --- | --- | --- | --- | --- | --- | --- | --- | --- | --- | --- | --- | --- | --- | --- | --- | --- | --- | --- | --- | --- | --- | --- |
|  |  |  | **Yes** | **No** |  | **Yes** | **No** |  | **Yes** | **No** |  | **Yes** | **No** |  | **Yes** | **No** |  | **Yes** | **No** |  | **Yes** | **No** |  | **Yes** | **No** |
| **Age** |  |  |  |  |  |  |  |  |  |  |  |  |  |  |  |  |  |  |  |  |  |  |  |  |  |
| Age＜65 | 7355  (43.2) |  | 772  (10.5) | 6583  (89.5) |  | 67  (0.9) | 7288  (99.1) |  | 23  (0.3) | 7332  (99.7) |  | 70  (1.0) | 7285  (99.0) |  | 259  (3.5) | 7096  (96.5) |  | 70  (1.0) | 7285  (99.0) |  | 70  (1.0) | 7285  (99.0) |  | 64  (0.9) | 7291  (99.1) |
| 65≤age<75 | 6706  (39.4) |  | 939  (14) | 5767  (86) |  | 71  (1.1) | 6635  (98.9) |  | 35  (0.5) | 6671  (99.5) |  | 99  (1.5) | 6607  (98.5) |  | 254  (3.8) | 6452  (96.2) |  | 81  (1.2) | 6625  (98.8) |  | 38  (0.6) | 6668  (99.4) |  | 61  (0.9) | 6645  (99.1) |
| Age≥75 | 2945  (17.4) |  | 426  (14.5) | 2519  (85.5) |  | 39  (1.3) | 2906  (98.7) |  | 30  (1.0) | 2915  (99.0) |  | 38  (1.3) | 2907  (98.7) |  | 107  (3.6) | 2838  (96.4) |  | 31  (1.1) | 2914  (98.9) |  | 16  (0.5) | 2929  (99.5) |  | 36  (1.2) | 2909  (98.8) |
| **Sex** |  |  |  |  |  |  |  |  |  |  |  |  |  |  |  |  |  |  |  |  |  |  |  |  |  |
| Female | 5414  (31.8) |  | 787  (14.5) | 4627  (85.5) |  | 67  (0.9) | 7288  (99.1) |  | 33  (0.6) | 5381  (99.4) |  | 52  (1.0) | 5362  (99.0) |  | 197  (3.6) | 5217  (96.4) |  | 63  (1.2) | 5351  (98.8) |  | 42  (0.8) | 5372  (99.2) |  | 40  (0.7) | 5374  (99.3) |
| Male | 11335  (66.7) |  | 2435  (21.5) | 8900  (78.5) |  | 71  (1.1) | 6635  (98.9) |  | 55  (0.5) | 11280  (99.5) |  | 153  (1.3) | 11182  (98.7) |  | 417  (3.7) | 10918  (96.3) |  | 117  (1.0) | 11218  (99.0) |  | 81  (0.7) | 11254  (99.3) |  | 121  (1.1) | 11214  (98.9) |
| Not specified | 257 (1.6) |  | 31  (12.1) | 226  (87.9) |  | 173  (1.0) | 16576  (99.0) |  | 0  (0.0) | 257  (100.0) |  | 2  (0.8) | 255  (99.2) |  | 6  (2.3) | 251  (97.7) |  | 2  (0.8) | 255  (99.2) |  | 1  (0.4) | 256  (99.6) |  | 0  (0.0) | 257  (100) |
| **Treatment modality** |  |  |  |  |  |  |  |  |  |  |  |  |  |  |  |  |  |  |  |  |  |  |  |  |  |
| Single | 15923  (93.6) |  | 3075  (19.3) | 12848  (80.7) |  | 169  (1.1) | 15502  (98.9) |  | 83  (0.5) | 15840  (99.5) |  | 190  (1.2) | 15733  (98.8) |  | 556  (3.5) | 15367  (96.5) |  | 169  (1.1) | 15754  (98.9) |  | 101  (0.6) | 15822  (99.4) |  | 156  (1.0) | 15767  (99.0) |
| Combinational | 1083  (6.3) |  | 178  (16.4) | 905  (83.6) |  | 4  (0.4) | 1074  (99.6) |  | 5  (0.5) | 1078  (99.5) |  | 17  (1.6) | 1066  (98.4) |  | 64  (5.9) | 1019  (94.1) |  | 13  (1.2) | 1070  (98.8) |  | 23  (2.1) | 1060  (97.9) |  | 5  (0.5) | 1078  (99.5) |
| **Comorbidity** |  |  |  |  |  |  |  |  |  |  |  |  |  |  |  |  |  |  |  |  |  |  |  |  |  |
| With | 16940  (99.6) |  | 3238  (19.1) | 13702  (80.9) |  | 173  (1.0) | 16510  (99.0) |  | 87  (0.5) | 16853  (99.5) |  | 207  (1.2) | 16733  (98.8) |  | 619  (3.7) | 16321  (96.3) |  | 181  (1.1) | 16759  (98.9) |  | 122  (0.7) | 16818  (99.3) |  | 159  (0.9) | 16781  (99.1) |
| Without | 66  (0.4) |  | 15  (22.7) | 51  (77.4) |  | 0  (0.0) | 66  (100) |  | 1  (1.5) | 65  (98.5) |  | 0  (0.0) | 66  (100.0) |  | 1  (1.5) | 65  (98.5) |  | 1  (1.5) | 65  (98.5) |  | 2  (3.0) | 64  (97.0) |  | 2  (3.0) | 64  (97.0) |

**Table *(continued)***

**Table *(continued)***

| **irAEs** **All**  **N=17006** | | | **Hypophysitis** | |  | **Encephalitis** | |  | **Skin reaction** | |  | **Diabetes** | |  | **Thyroid toxicity** | |  | **Hematologic toxicity** | |  | **Neurologic toxicity** | |  |
| --- | --- | --- | --- | --- | --- | --- | --- | --- | --- | --- | --- | --- | --- | --- | --- | --- | --- | --- | --- | --- | --- | --- | --- |
|  |  |  | **Yes** | **No** |  | **Yes** | **No** |  | **Yes** | **No** |  | **Yes** | **No** |  | **Yes** | **No** |  | **Yes** | **No** |  | **Yes** | **No** |  |
| **Age** |  |  |  |  |  |  |  |  |  |  |  |  |  |  |  |  |  |  |  |  |  |  |  |
| Age＜65 | 7355  (43.2) |  | 43  (0.6) | 7312  (99.4) |  | 67  (0.9) | 7288  (99.1) |  | 133  (1.8) | 7222  (98.2) |  | 109  (1.5) | 7246  (98.5) |  | 66  (0.9) | 7289  (99.1) |  | 67  (0.9) | 7288  (99.1) |  | 12  (0.2) | 7343  (99.8) |  |
| 65≤age<75 | 6706  (39.4) |  | 35  (0.5) | 6671  (99.5) |  | 58  (0.9) | 6648  (99.1) |  | 114  (1.7) | 6592  (98.3) |  | 127  (1.9) | 6579  (98.1) |  | 46  (0.7) | 6660  (99.3) |  | 71  (1.1) | 6635  (98.9) |  | 9  (0.1) | 6697  (99.9) |  |
| Age≥75 | 2945  (17.4) |  | 7  (0.2) | 2938  (99.8) |  | 28  (1.0) | 2917  (99.0) |  | 55  (1.9) | 2890  (98.1) |  | 52  (1.8) | 2893  (98.2) |  | 20  (0.7) | 2925  (99.3) |  | 39  (1.3) | 2906  (98.7) |  | 1  (0.0) | 2944  (100) |  |
| **Sex** |  |  |  |  |  |  |  |  |  |  |  |  |  |  |  |  |  |  |  |  |  |  |  |
| Female | 5414  (31.8) |  | 25  (0.5) | 5389  (99.5) |  | 65  (1.2) | 5349  (98.8) |  | 120  (2.2) | 5294  (97.8) |  | 81  (1.5) | 5333  (98.5) |  | 66  (0.9) | 7289  (99.1) |  | 67  (0.9) | 7288  (99.1) |  | 12  (0.2) | 7343  (99.8) |  |
| Male | 11335  (66.7) |  | 59  (0.5) | 11276  (99.5) |  | 88  (0.8) | 11247  (99.2) |  | 179  (1.6) | 11156  (98.4) |  | 206  (1.9) | 11129  (98.2) |  | 46  (0.7) | 6660  (99.3) |  | 71  (1.1) | 6635  (98.9) |  | 9  (0.1) | 6697  (99.9) |  |
| Not specified | 257 (1.6) |  | 1  (0.4) | 256  (99.6) |  | 0  (0.0) | 257  (100.0) |  | 299  (1.8) | 16450  (98.2) |  | 287  (1.7) | 16462  (98.3) |  | 803  (4.8) | 15946  (95.2) |  | 173  (1.0) | 16576  (99.0) |  | 21  (0.1) | 16728  (99.9) |  |
| **Treatment modality** |  |  |  |  |  |  |  |  |  |  |  |  |  |  |  |  |  |  |  |  |  |  |  |
| Single | 15923  (93.6) |  | 65  (0.4) | 15858  (99.6) |  | 135  (0.8) | 15788  (99.2) |  | 290  (1.9) | 15381  (98.1) |  | 259  (1.7) | 15412  (98.3) |  | 763  (4.9) | 14908  (95.1) |  | 169  (1.1) | 15502  (98.9) |  | 21  (0.1) | 15650  (99.9) |  |
| Combinational | 1083  (6.3) |  | 20  (1.8) | 1063  (98.2) |  | 18  (1.7) | 1065  (98.3) |  | 9  (0.8) | 1069  (99.2) |  | 28  (2.6) | 1050  (97.4) |  | 40  (3.7) | 1038  (96.3) |  | 4  (0.4) | 1074  (99.6) |  | 0  (0.0) | 1078  (100.0) |  |
| **Comorbidity** |  |  |  |  |  |  |  |  |  |  |  |  |  |  |  |  |  |  |  |  |  |  |  |
| With | 16940  (99.6) |  | 85  (0.5) | 16855  (99.5) |  | 153  (0.9) | 16787  (99.1) |  | 299  (1.8) | 16384  (98.2) |  | 285  (1.7) | 16398  (98.3) |  | 798  (4.8) | 15885  (95.2) |  | 173  (1.0) | 16510  (99.0) |  | 21  (0.1) | 16662  (99.9) |  |
| Without | 66  (0.4) |  | 0  (0.0) | 66  (100.0) |  | 0  (0.0) | 66  (100) |  | 0  (0.0) | 66  (100) |  | 2  (3.0) | 64  (97.0) |  | 5  (7.6) | 61  (92.4) |  | 0  (0.0) | 66  (100) |  | 0  (0.0) | 66  (100) |  |

Notes: irAEs: Immune-related adverse events; Yes: With irAEs; No: Without irAEs.
